# Effect of cellular senescence on the response of human peritoneal mesothelial cells to TGF-β

**DOI:** 10.1038/s41598-024-63250-1

**Published:** 2024-06-03

**Authors:** Edyta Kawka, Rebecca Herzog, Marcin Ruciński, Agnieszka Malińska, Markus Unterwurzacher, Juan Manuel Sacnun, Anja Wagner, Katarzyna Kowalska, Karol Jopek, Agata Kucz-Chrostowska, Klaus Kratochwill, Janusz Witowski

**Affiliations:** 1 Department of Pathophysiology, Poznan University of Medical Sciences, Poznan, Poland; 2 Christian Doppler Laboratory for Molecular Stress Research in Peritoneal Dialysis, Medical University of Vienna, Vienna, Austria; 3 Division of Pediatric Nephrology and Gastroenterology, Department of Pediatrics and Adolescent Medicine, Comprehensive Center for Pediatrics, Medical University of Vienna, Vienna, Austria; 4 Department of Histology and Embryology, Poznan University of Medical Sciences, Poznan, Poland

**Keywords:** Mesothelial cells, Mesothelial-to-mesenchymal transition, Cellular senescence, TGF-β, Cell signalling, Senescence, Renal replacement therapy

## Abstract

Transforming growth factor β (TGF-β) is implicated in both mesothelial-to-mesenchymal transition (MMT) and cellular senescence of human peritoneal mesothelial cells (HPMCs). We previously showed that senescent HPMCs could spontaneously acquire some phenotypic features of MMT, which in young HPMCs were induced by TGF-β. Here, we used electron microscopy, as well as global gene and protein profiling to assess in detail how exposure to TGF-β impacts on young and senescent HPMCs in vitro. We found that TGF-β induced structural changes consistent with MMT in young, but not in senescent HPMCs. Of all genes and proteins identified reliably in HPMCs across all treatments and states, 4,656 targets represented overlapping genes and proteins. Following exposure to TGF-β, 137 proteins and 46 transcripts were significantly changed in young cells, compared to 225 proteins and only 2 transcripts in senescent cells. Identified differences between young and senescent HPMCs were related predominantly to wound healing, integrin-mediated signalling, production of proteases and extracellular matrix components, and cytoskeleton structure. Thus, the response of senescent HPMCs to TGF-β differs or is less pronounced compared to young cells. As a result, the character and magnitude of the postulated contribution of HPMCs to TGF-β-induced peritoneal remodelling may change with cell senescence.

## Introduction

A monolayer of mesothelial cells lines the peritoneal membrane that covers the walls and internal organs of the abdominal cavity. The morphology of human peritoneal mesothelial cells (HPMCs) resembles that of classical epithelium, with a characteristic cobblestone appearance. HPMCs can also adopt a fibroblast-like phenotype by undergoing epithelial-to-mesenchymal transition. However, given the mesodermal rather than epithelial origin of mesothelial cells, this process is usually referred to as mesothelial-to-mesenchymal transition (MMT)^1^. It is associated with changes in cell signalling programmes that control the disassembly of intercellular junctions, reorganization of the cytoskeleton, loss of apical–basal polarity, and degradation of the basement membrane, resulting in HPMCs losing their epithelial-like features and acquiring the phenotypic and functional properties of myo-fibroblasts^2^. These changes include a gradual loss of E-cadherin expression and increased expression of α-smooth muscle actin (α-SMA), as well as extracellular matrix (ECM) proteins and matrix metalloproteases. Such features enable HPMCs to migrate from the monolayer to the underlying stroma and deposit ECM components^3^. The process of MMT occurs during embryonic development^4^, but also in pathologies affecting the peritoneum, including peritoneal adhesions^5^, endometriosis^6^, ovarian cancer^7–9^, and peritoneal dialysis (PD)-associated fibrosis^10^.

Transforming growth factor-β (TGF-β) has been identified as a key driver of MMT in vitro and in vivo^2,11–13^. TGF-β has pleiotropic effects both in health and disease (see ^14,15^ for recent reviews). TGF-β exerts its functions in a manner that is highly dependent on the pathophysiological context^16^. Among others, it has been shown that TGF-β can promote cellular senescence of fibroblasts^17^, bronchial epithelial cells^18,19^, or some cancer cells^20^.

Cellular senescence is a complex form of cell response to stress that jeopardizes genome integrity. The known triggers of senescence include telomere dysfunction, oncogene activation, reactive oxygen species, and epigenomic damage^21,22^. The resulting cellular senescence is characterized by irreversible growth arrest, distorted cellular morphology, resistance to apoptosis, and altered gene expression profiles^23,24^. Despite these changes, senescent cells remain metabolically active and produce increased quantities of various compounds—a feature known as the senescence-associated secretory phenotype (SASP)^25,26^. SASP components include numerous cytokines, chemokines, growth factors, proteases, and matrix proteins, through which senescent cells exert a strong paracrine effect on neighbouring cells and the tissue microenvironment^27,28^.

Only a few studies analysed the transcriptome and proteome of mesothelial cells exposed to TGF-β. However, these studies involved only young cells isolated freshly from humans^29,30^ or rats^31^, while similar studies on senescent HPMCs are lacking. We have previously observed that senescent HPMCs spontaneously acquire some features of MMT, including the expression of α-SMA and fibroblast-specific protein 1 (FSP1)^32^. The mechanism of this effect is not fully understood, but it is linked to up-regulation of Snail, a key transcriptional regulator of MMT^1^. Also, the observation that senescent HPMCs with MMT features do not completely lose their epithelial-like characteristics and appear less susceptible to the development of MMT in response to TGF-β remains to be elucidated^32^. Therefore, we aimed to extend these observations and used electron microscopy, transcriptomics, and proteomics to identify differences between young and senescent cells in their responsiveness to TGF-β.

## Results

### Effect of TGF-β on the morphology and ultrastructure of young and senescent HPMCs

**Figure 1:**
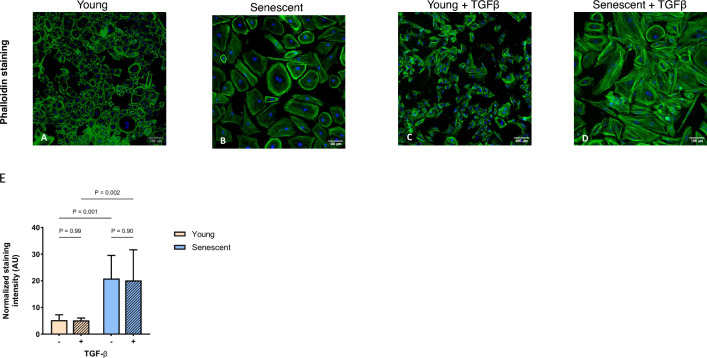
Morphology of young and senescent HPMCs treated with TGF-β. Cells were stained with phalloidin (green) and DAPI (blue); (**A**) young HPMCs, (**B**) senescent HPMCs, (**C**) young HPMCs treated with TGF-β, (**D**) senescent HPMCs treated with TGF-β; magnification 200 ×, scale bar 100 μm. (**E**) The fluorescence intensities of phalloidin staining normalized to nuclear staining was quantified by ImageJ software and analyzed with two-way ANOVA. The data are represented as means (± SD) from 3 to 6 images per condition.

**Figure 2:**
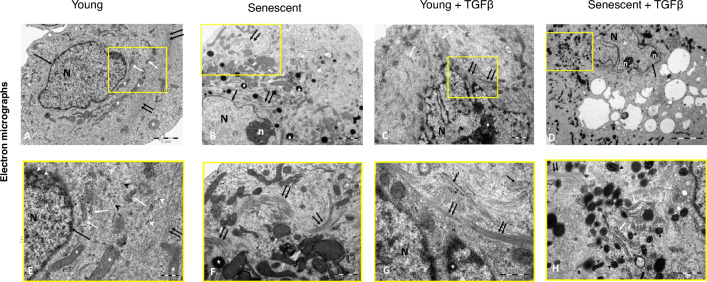
Electron microphotographs of HPMCs. (**A** and **E**) Young HPMCs—centrally located disc-shaped nucleus (*N*) with a slightly irregular heterochromatin outline (*black arrow*); short rough endoplasmic reticulum cisterns (*white arrows*), free ribosomes (*black arrowheads*) and polyribosomes (*white arrowheads*); mitochondria with densely packed long cristae and an electron-dense mitochondrial matrix (*white asterisks*); microfilaments forming a ring around the nucleus (*double black arrows*). An inset (**E**) also shows cross-sections of pores in the nuclear envelope (*double white arrows*). Scale bars correspond to 2 μm (**A**) and 500 nm (**E**). (**B** and **F**) Senescent HPMCs—euchromatic cell nucleus (*N*) with a fairly regular outline and a narrow band of heterochromatin (*black arrow*) close to the nuclear envelope, and with a nucleolus (*n*); mitochondria (*white arrows*) with an electron-dense matrix and loosely packed short mitochondrial cristae; vimentin filaments both in the vicinity of the nucleus and in peripheral regions (*double arrows*); multiple lipid droplets (*asterisks*). Scale bars correspond to 1 μm (**B**) and 500 nm (**F**). (**C** and **G**) Young HPMCs after exposure to TGF-β—dense bundles of vimentin filaments (*double black arrows*) around the nucleus (*N*) and at one of the cell poles (*white arrows*); cell nucleus with an irregular outline and many invaginations, and abundant electron-dense heterochromatin (*asterisk*). An inset (**G**) also shows rough endoplasmic reticulum cisterns (*black arrows*). Scale bars correspond to 1 μm (**C**) and 500 nm (**G**). (**D** and **H**) Senescent HPMCs after exposure to TGF-β—cell nucleus (*N*) rich in euchromatin with an irregular outline and narrow band of heterochromatin near the nuclear envelope (*black arrow*), and containing inclusion bodies (*white asterisk*) and nucleoli (*n*); pleomorphic mitochondria (*black arrowheads*) with an electron-dense matrix; rough endoplasmic reticulum cisterns (*white arrows*); vimentin filaments (*double black arrows*). Scale bars correspond to 5 μm (D) and 1 μm (H).

Consistent with previous observations^32^, senescent HPMCs had a distinctly different phenotype than young cells. After staining the cytoskeleton with phalloidin, young HPMCs showed a typical cobblestone appearance, unlike senescent cells which appeared rounded and greatly enlarged (Fig. 1A and B). As a result, the abundance of actin filaments in senescent cells was significantly greater than in young cells (Fig. 1E). Differences between young and senescent HPMCs were also seen at the ultrastructural level (Fig. 2A,B and E,F). In young HPMCs, the centrally located discoid nucleus was surrounded by mitochondria and endoplasmic reticulum, whose features did not point to intense cell activity. They included few short cisternae of rough endoplasmic reticulum, free ribosomes and mitochondria with densely packed long cristae and an electron-dense mitochondrial matrix. Further outwards, the microfilaments formed a ring around the nucleus.

In contrast, in senescent cells, mitochondria with an electron-dense matrix were scattered throughout the cytoplasm and had rather loosely packed short cristae. Moreover, these cells contained numerous lipid droplets as judged by their opalescent appearance in cross-sections and the absence of a surrounding cell membrane. Microfilaments were present not only in the vicinity of the nucleus, but also in peripheral areas.

After exposure to TGF-β, young HPMCs lost their cobbled morphology and acquired a spindle-shape appearance (Fig. 1C). Although the overall intensity of staining for actin filaments did not change significantly (Fig. 1E), electron microscopy revealed pronounced changes in the distribution of vimentin microfilaments, whose dense bundles were visible not only around the nucleus, but also at one of the cell poles (Fig. 2C and G). The nucleus became irregular and rich in electron-dense heterochromatin.

Senescent cells exposed to TGF-β were equally enlarged as untreated cells and even more elongated (Fig. 1D). As with young cells, exposure of senescent cells to TGF-β did not significantly change the total amount of stained actin filaments (Fig. 1E). Electron microscopy confirmed extensive cellular hypertrophy, and general ultrastructural features were similar to those found in untreated senescent cells. Interestingly, however, numerous mitochondria present in the cytoplasm exhibited features of both polymorphism and pleomorphism (Fig. 2D and H).

### Effect of TGF-β on the transcriptome of young and senescent HPMCs

**Figure 3:**
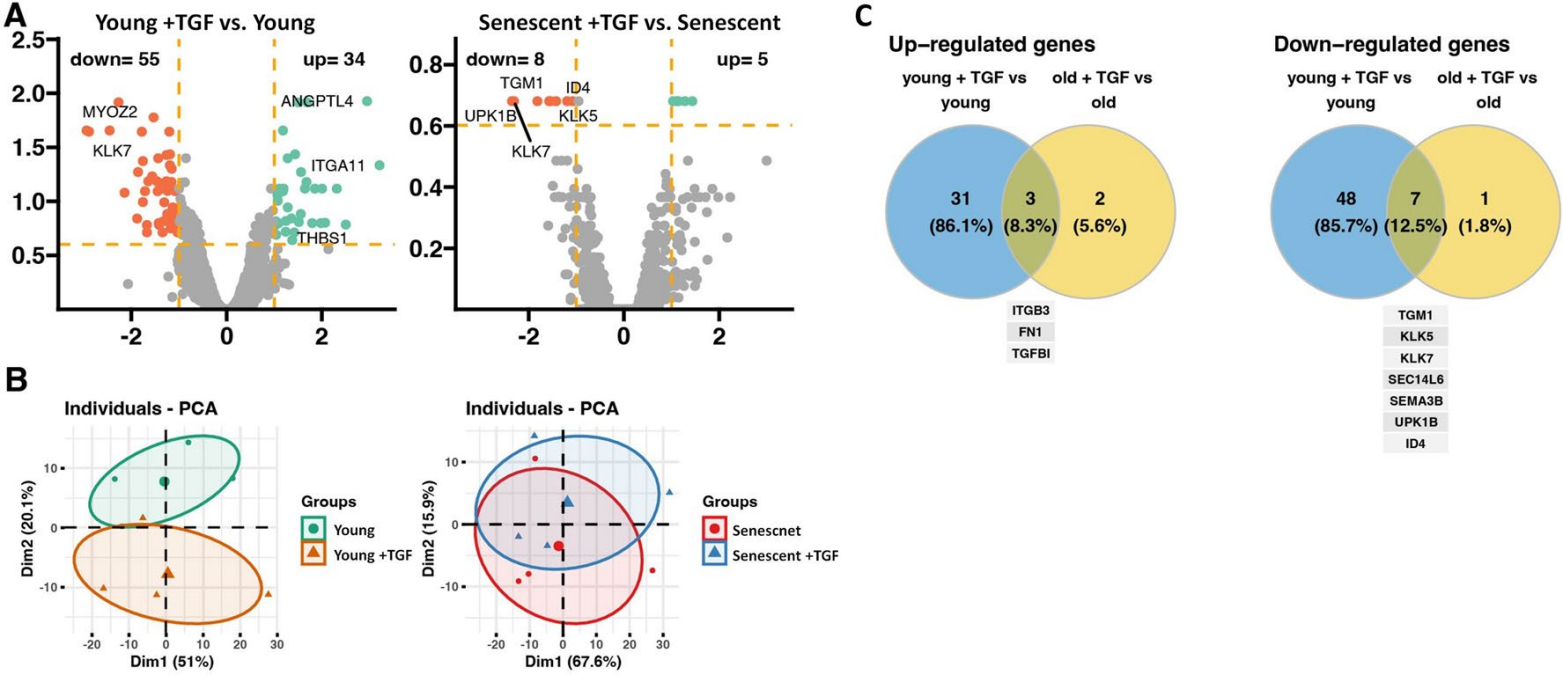
Transcriptome regulation by TGF-β in young and senescent cells. (**A**) Volcano plots of gene expression profiles in young and senescent cells treated with TGF-β. Each data point corresponds to the mean normalized expression level of a specific gene from four individual donors. The orange dashed lines are placed at cut-off values based on the following parameters: |fold change|> 2 and *p*-value < 0.05 with 25% FDR. Genes that fulfilled the selection criteria are considered differentially expressed (DEGS) and are represented as red (down-regulated) and green (up-regulated) dots. Total number of DEGS are presented on the left and right upper corners of the graph. The five most regulated genes are marked by their gene symbols. (**B**) Principal component analysis (PCA) plots showing the first two principal components of the filtered microarray data set. (**C**) Venn diagrams illustrating the overlap and unique genes that undergo up- and down-regulation across all analysed groups compared to untreated cells. Symbols of genes common for the groups compared are shown.

Overall, the microarray analysis identified 26,842 unique transcripts, of which 6,803 had abundance values in all samples from 4 separate donors, and which were used for further analyses (Supplemental Table S1). Global gene expression profiles in TGF-β-treated cells were compared with those in untreated controls and first presented as volcano plots (Fig. 3A). Exposure of young HPMCs to TGF-β resulted in significant up-regulation of 34 genes and down-regulation of 55 genes, whereas in senescent HPMCs only 5 genes showed significantly increased expression and 8 genes showed decreased expression (*p* < 0.05). The Principal Component Analysis (PCA) with top 500 regulated genes revealed differences in the datasets across experimental groups (Fig. 3B). The datasets from TGF-β-treated and untreated young HPMCs were found to be more distinct, while the clusters from senescent cells overlapped significantly in this respect.

Similarities between the groups in the expression of individual genes were then analysed, as shown in the Venn diagram (Fig. 3C). It turned out that after TGF-β treatment, the expression of only 10 genes changed consistently in both young and senescent cells. Of these genes, 3 were found to be up-regulated and 7 down-regulated.

Genes that were identified in young and senescent HPMCs as differentially expressed after TGF-β treatment (absolute fold change > 2 and p < 0.05 with 25% FDR correction) were subjected to ontology group enrichment analysis using the DAVID database. However, due to the small number of genes differentially expressed in senescent cells, significantly enriched ontological groups could be consistently identified only in young cells (Supplementary Fig. S1).

**Figure 4:**
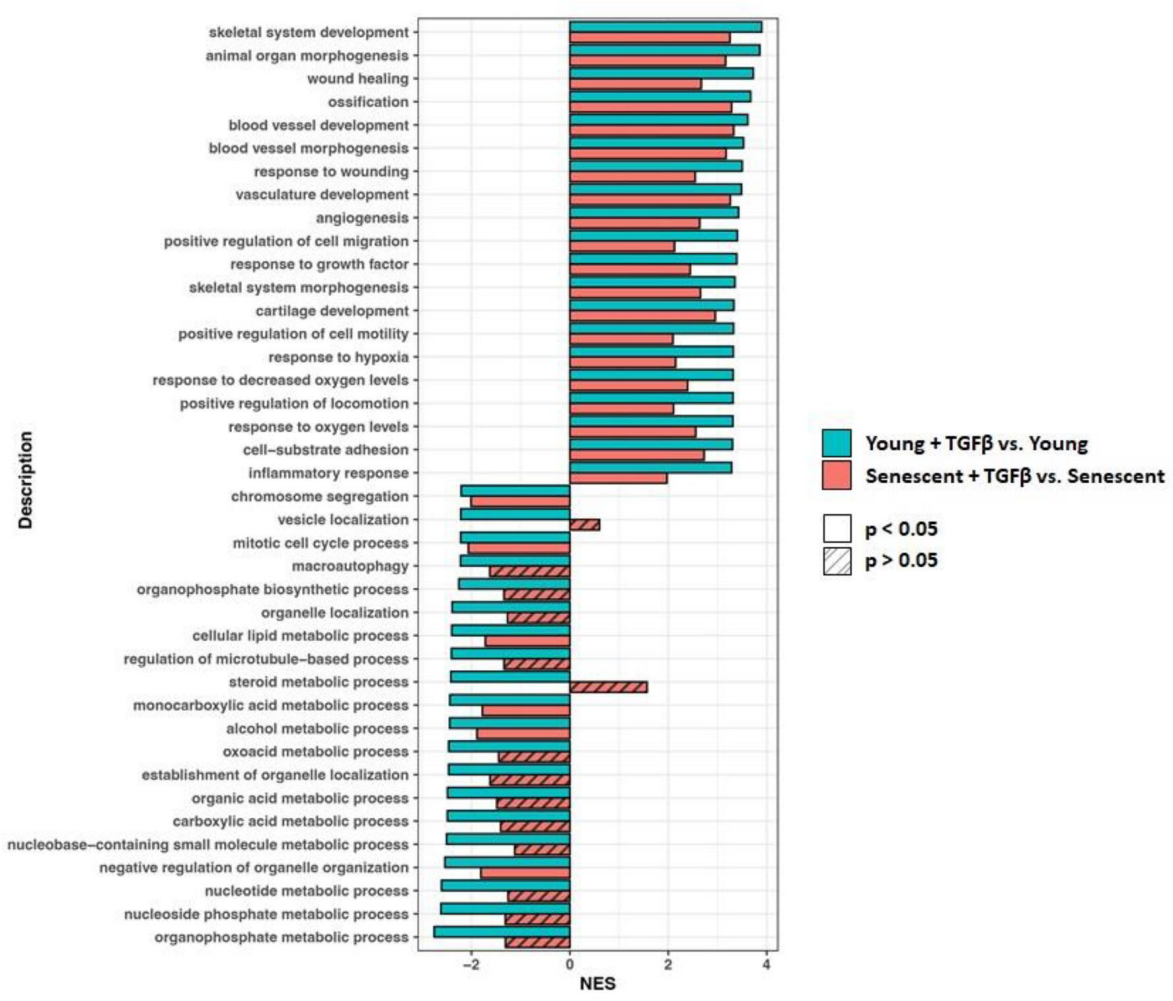
Gene set enrichment analysis. The response to TGF-β was compared between young cells (green) and senescent cells (red). Bars represent twenty ontological groups with the highest (activated processes—positive values) and lowest (inhibited processes—negative values) values of normalized enrichment score (NES).

The regulation of ontological processes by TGF-β was assessed using Gene Set Enrichment Analysis (GSEA), and revealed that exposure to TGF-β resulted in prominent enrichment of genes involved in processes categorized broadly as skeletal development, wound healing, angiogenesis, cell motility, inflammation, and response to growth factors and response to hypoxia (Fig. 4). GSEA also identified down regulated groups of genes associated with microtubule-mediated cellular organization, organelle localization and cell cycle progression, as well as with metabolism of nucleotide components and organic acids. These effects were seen both in young and senescent HPMCs, however, compared to senescent cells, the absolute values of normalized enrichment score (NES) for identified processes were consistently higher in young HPMCs.

### Effect of TGF-β on the proteome of young and senescent HPMCs

**Figure 5:**
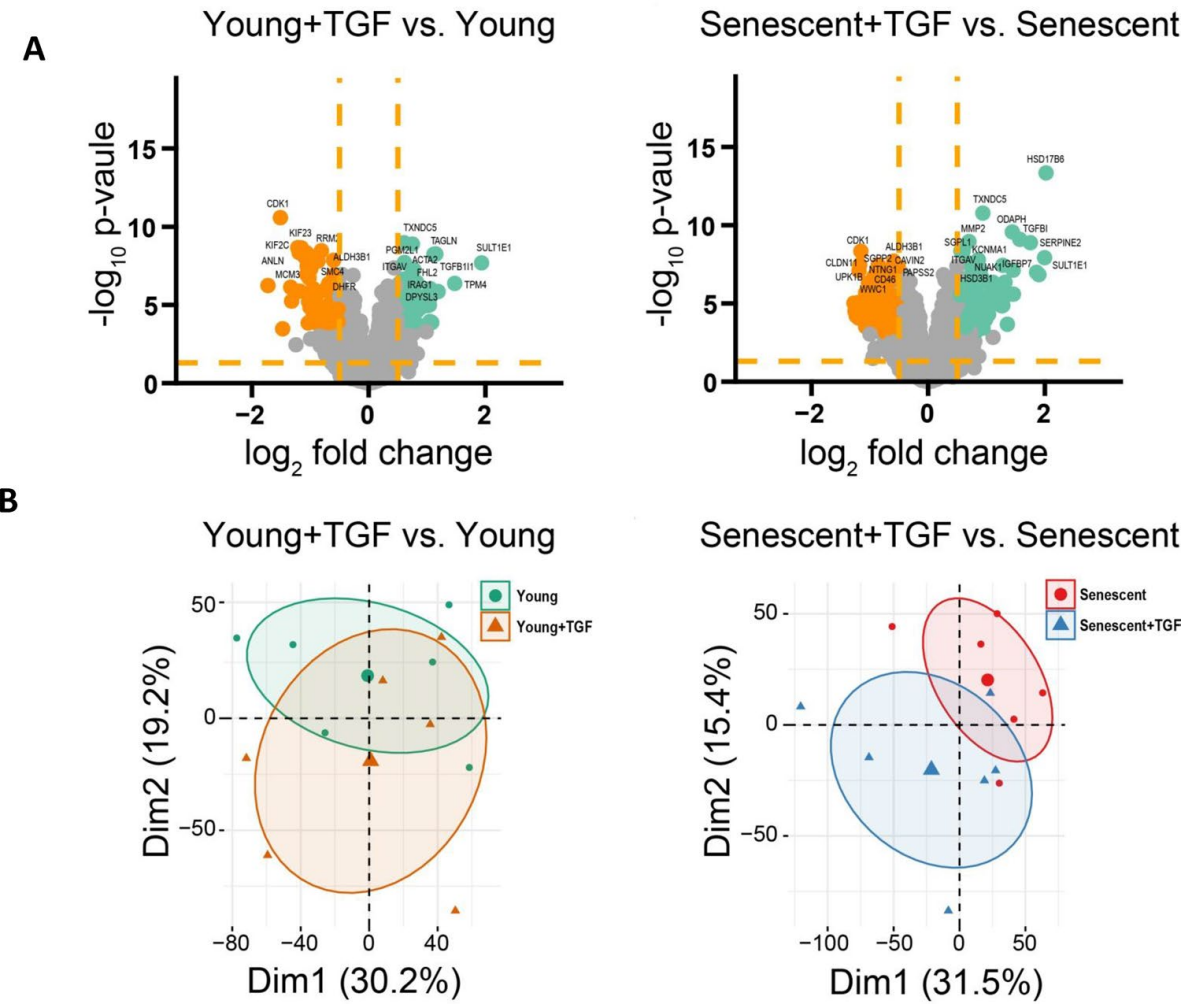
Proteome regulation by TGF-β in young and senescent cells. (**A**) Volcano plots for regulated proteins in young and senescent cells treated with TGF-β. Each data point corresponds to the mean normalized expression level of a specific protein from six individual donors. *p* < 0.05 for all points above dashed line, coloured points are *p* < 0.01 (LIMMA) after Benjamini-Hochberg (BH) correction and log2 [fold change] > 0.5. (**B**) PCA plots showing first two principal components of the filtered, top 500 regulated proteins of the proteomics data set.

To investigate the effects of TGF-β on the HPMCs proteome, a multiplex bottom-up proteomics approach, based on isobaric labelling of digested proteins, off-line fractionation, and high-resolution mass spectrometry, was applied. After excluding single-peptide identifications and removing contaminants, 8,781 unique proteins were identified to have abundance values in all 24 samples from 6 individual donors and were used for further analysis (Supplemental Table S2). Changes in the proteome are presented as volcano plots (Fig. 5A). The analysis revealed that in young HPMCs treated with TGF-β the expression of 131 proteins was significantly changed, with 72 proteins down-regulated and 59 proteins up-regulated, whereas in senescent cells, the expression of 232 proteins was significantly altered, with 127 proteins down-regulated and 105 proteins up-regulated (*p* < 0.01 after Benjamini-Hochberg (BH) correction and log2 [fold change] > 0.5). The proteins most affected by TGF-β1 exposure in young and senescent cells are listed in Supplemental Table S3. The PCA with the top 500 regulated proteins showed no clear separation of TGF-β-treated and untreated HPMCs in both young and senescent cells, however the sets from senescent cells appeared to be more distinct (Fig. 5B).

**Figure 6:**
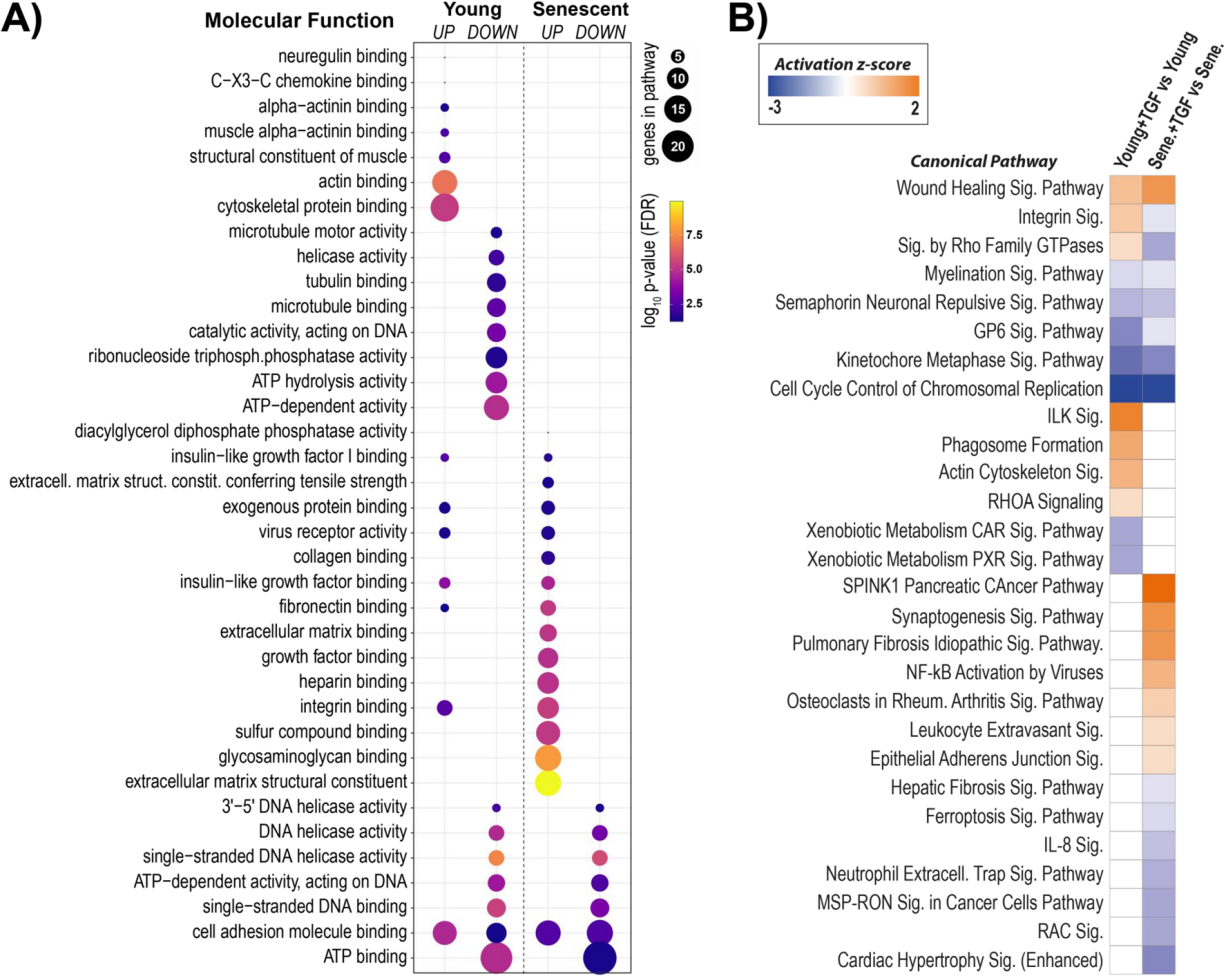
Pathway analysis of proteins significantly regulated by TGF-β. (**A**) Bubble plot of enriched GO molecular functions. (**B**) Activation heatmap of enriched canonical pathways from ingenuity pathway analysis (IPA, Fisher’s exact test, *p* < 0.05) for the effect of TGF-β in young and senescent HPMCs.

Protein enrichment analysis revealed potential differences between young and senescent cells in TGF-β-regulated pathways classified according to molecular function (Fig. 6A), cellular compartment, and biological process (Supplemental Fig. S2A and S2B). While all molecular functions downregulated by TGF-β in senescent cells were also downregulated in young cells, only 50% of the molecular functions enriched by TGF-β in senescent cells were up-regulated also in young cells. In addition, there was a number of molecular functions affected by TGF-β only in young cells. A canonical pathway overrepresentation analysis (*p* < 0.05) not only identified pathways affected by TGF-β both in young and senescent HPMCs but also assessed them in terms of their activation status (Fig. 6B). These included the processes of wound healing (activated in both cases), cell cycle control and kinetochore assembly, and GP6 signalling for collagen binding (inhibited in both cases). Interestingly, TGF-β also affected pathways related to signalling through integrins and the Rho family of GTPases, but seemed to activate them in young cells and inhibit them in senescent cells.

### Integration of transcriptomic and proteomic data

**Figure 7:**
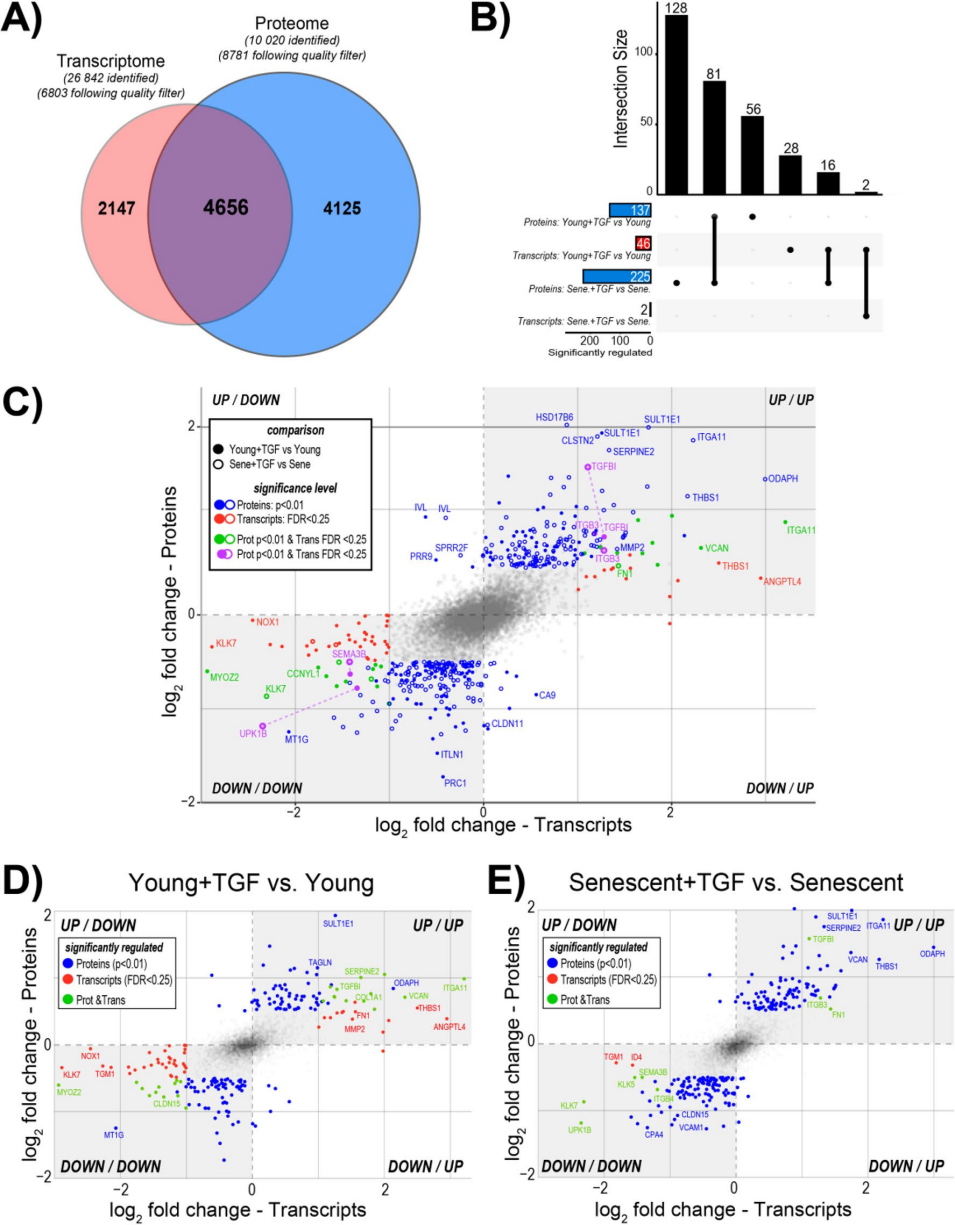
Integration of transcriptomics and proteomics data. (**A**) Venn diagram illustrating the overlap of identified proteins with identified transcripts. (**B**) Upset R plot illustrating the individual overlap of significantly regulated proteins and transcripts in each condition. The stacked bars on the left (significantly regulated) display the total number of proteins and transcripts significantly regulated in each condition. The bar plot at the top shows the number of proteins or transcripts in each comparisons or condition as indicated by the matrix below each bar. (**C**) Co-regulation analysis of genes and proteins significantly regulated (*p* < 0.05) in young (filled dots) and senescent (open dots) cells after treatment with TGF-β. Blue—significant (*p* < 0.01) at protein level, red—significant (FDR < 0.25) at transcriptome level, green—significant at protein and transcriptome level in either comparisons, purple—significant on protein and transcript level in both comparisons. (**D** and **E**) Co-regulation analysis of genes and proteins significantly regulated (*p* < 0.05) in young (**D**) and senescent (**E**) cells after treatment with TGF-β. Blue—significant (*p* < 0.01) at protein level, red—significant (FDR < 0.25) at transcriptome level, green—significant at protein and transcriptome level in either comparisons.

Of the 6,803 transcripts and 8,781 proteins over all treatments and cell states, we found 4,656 genes/proteins in both analyses, accounting for 68% of the quantified genes and 53% of the quantified proteins (Fig. 7A). Following TGF-β exposure, 137 proteins and 46 transcripts were found to be significantly changed in young cells, while 225 proteins but only 2 transcripts were found to be changed in senescent cells following TGF-β. (Fig. 7B). The responses to TGF-β identified at both the transcriptome and protein levels and present in both young and senescent HPMCs included TGFBI (TGF-β-induced) and ITGB3 (β3 integrin), which were upregulated, and UPK1B (uroplakin 1B) and SEMA3B (semaphorin 3B), which were downregulated (Fig. 7C)**.** Changes in response to TGF-β seen at the RNA and protein level included also VCAN (versican), ITGA11 (integrin α11), FN1 (fibronectin 1), CCNYL1 (cyclin Y-like 1) and MYOZ2 (myozenin 2), however, these were observed only in young cells. A decrease in KLK7 (kallikrein 7) gene expression after exposure to TGF-β was seen both in young and senescent HPMCs, with a decrease in KLK7 protein detected only in senescent cells. Finally, exposure to TGF-β led to an increase in THBS1 (thrombospondin 1) expression in young cells and at the protein level in senescent cells (Fig. 7D,E).

## Discussion

TGF-β is thought to be the main driver of mesenchymal transition of epithelial and mesothelial cells. In this respect, it shows powerful activity both on its own and in co-operation with other cytokines (reviewed in^33^). Numerous studies investigated the TGF-β-induced MMT of apparently young cells^10,29,31^. However, our earlier observations suggested that the effect of TGF-β can be different in senescent cells^32^. To gain a more detailed insight into how TGF-β affects young versus senescent HPMCs, in the present study, we compared changes in the ultra-structure as well as in the transcriptomic and proteomic profiles of young and senescent HPMCs exposed to TGF-β. We observed some similarities, but also large differences in the direction and magnitude of alterations induced by TGF-β.

As expected, exposure of young HPMCs to TGF-β for 72 h resulted in changes in cell phenotype consistent with MMT^1,32^. The acquirement of a fibroblast-like appearance by HPMCs was associated with ultrastructural re-organization of the cytoskeletal filaments. These cells also showed significant differences in the expression of 89 genes and 131 proteins. Of those, approximately 60% were found to be down-regulated. This is in line with a previous observation suggesting that TGF-β-induced MMT in vitro is largely a repression process^30^. Accordingly, enrichment analysis revealed that under-represented sets of genes and proteins in TGF-β-treated young HPMCs were those involved in cytoskeleton organization, DNA processing, cell cycle progression and nucleotide metabolism. These effects correspond with the view that in normal epithelial cells TGF-β acts as an inhibitor of cell proliferation^34^. On the other hand, sets of genes and proteins that were over-represented in TGF-β-treated young HPMCs pointed to increased activity of pathways involved in wound healing and angiogenesis. This is in keeping with the common perception of TGF-β as a master regulator of wound healing and fibrosis^15^.

In contrast to distinct changes in cytomorphology induced by TGF-β in young HPMCs, the effect of TGF-β on senescent cells was rather small and more difficult to recognize. Of note, senescent HPMCs treated with TGF-β were found to have highly pleomorphic mitochondria. It can be speculated that this reflected mitochondrial fusion aiming to maintain a pool of larger and “healthier” mitochondria in cells, whose mitochondrial biogenesis, dynamics and function was compromised by aging^35^. In addition, the number of genes expressed differentially upon TGF-β exposure in senescent cells was lower than in young HPMCs. Although the processes identified by GSEA as regulated by TGF-β in young cells were also detected in senescent cells, they had lower normalized enrichment scores (NES). There were only a few processes with higher NES in senescent cells and these were associated with microtubule-based movement and protein localization to membranes.

Interestingly, compared to young cells, exposure to TGF-β changed the expression of almost twice as many proteins in senescent cells. One may wonder whether this is related to the increased size of senescent cells. In this respect, Lanz et al. observed that when cells grew larger, the concentrations of many individual and highly abundant proteins in the proteome increased to a greater extent than cell size^36^. Pathway analysis based on proteins enriched after TGF-β exposure indicated that the regulation of some processes (e.g. wound healing or cell cycle control) was similar in young and senescent cells. The other processes, however, seemed to be regulated differently, as exemplified by signalling through integrins, which appeared to be stimulated in young cells, but repressed in senescent ones. In this respect, using young HPMCs stimulated with TGF-β, Han et al. have previously reported on significantly enriched expression of genes involved in integrin binding^29^. Interestingly, we found that expression of ITGA11, the gene encoding the subunit alpha 11 of integrins, increased in response to TGF-β significantly more in young HPMCs than in senescent cells. Integrins are key cellular receptors that mediate interactions between extracellular matrix and cytoskeleton and impact on cell phenotype and function. The α11-containing integrin α11β1 that preferentially binds to collagen I was shown to be stimulated by TGF-β in fibroblasts and involved in organ fibrosis^37,38^. In this respect, it has been reported that ITGA11 depletion reduced the development of myo-fibroblastic features in cells stimulated with TGF-β and alleviated liver fibrosis^38^. This finding may correspond to our previous observations suggesting that cellular senescence may limit the development of a full-blown fibroblastic phenotype in HPMCs^32^. In this respect, it has been demonstrated that senescence of hepatic stellate cells protects against excessive fibrosis following acute liver injury in mice^39^. Moreover, mesothelial cells covering the capsule of the liver were found to undergo MMT and differentiate into stellate cells in a TGF-β-dependent manner^40^. Intriguingly, we found that the protein profile of senescent HPMCs pointed to reduced activity of the liver fibrosis signalling pathway.

Another gene and protein that was upregulated by TGF-β to a greater extent in young than in senescent cells was thrombospondin-1 (THBS1). Increased expression of ITGA11 and THBS1 in young cells upon TGFβ stimulation were also observed in genome microarray analysis of human peritoneal cells that undergone, or were during MMT^30^ and RNA-seq data^31^. THBS1 is a multi-functional protein, whose increased expression was observed in tissue injury and repair, cellular senescence and ageing-related diseases^41–44^. THBS1 is thought to mediate TGF-β activation by dissociating it from the latency-associated peptide^45^. Thus, the stimulation of THSB1 by TGF-β may create a fast-forward loop that promotes TGF-β functions in young cells. In contrast, reduced THBS1 expression would limit the effect of TGF-β in senescent cells. Moreover, THBS1 can exert TGFβ-independent effects that contribute to the regulation of haemostasis, cell adhesion, migration, growth factor activity, and angiogenesis^42^. For example, it has been demonstrated that while full length THBS1 inhibits angiogenesis by inducing apoptosis of endothelial cells^46^, THBS1 cleavage by kallikrein-related peptidase 7 (KLK7) creates a 28 kDa N-terminal fragment that promotes angiogenesis^47^. In this respect, we found that TGF-β decreased expression of KLK7 in both young and senescent HPMCs, which could potentially prevent THBS1 from cleaving and result in reduced angiogenesis. Although we have previously observed that TGF-β is a powerful inducer of vascular endothelial growth factor (VEGF) in young HPMCs^48^, the transcriptomic and proteomic analysis in the present study did not reveal a significantly different response of senescent cells in this regard.

Pathway analysis using the IPA tool pointed to cytoskeleton regulation by TGF-β in young and senescent cells by revealing the activation of actin and RhoA signalling in these cells. On the other hand, the same analysis identified activation of serine peptidase inhibitor Kazal type I (SPINK1) pancreatic cancer pathway and leukocyte extravasation signalling in senescent but not in young HPMCs. The role of SPINK1 in health and disease is of great interest because SPINK1 activity can exert both beneficial and harmful effects, with aberrant SPINK1 signalling being strongly associated with cancer progression. However, the molecular biology associated with SPINK1 signalling in a specific tissue context is not fully understood^49,50^. Therefore, our observation of an apparently increased activity of the SPINK1 pathway in senescent HPMCs is difficult to interpret. Nevertheless, it may be consistent with a concept of senescence being an anti-tumour mechanism, but also promoting the development of age-related malignancies^51,52^. The latter is largely related to the senescence-associated secretory phenotype that involves an increased production of pro-inflammatory cytokines^23^. These may drive leukocyte extravasation, tissue infiltration and chronic inflammation that alters tissue architecture and paves the way for diseases of ageing^53^. In this respect, leukocyte extravasation signalling pathway was found to be an important part of analysed senescence secretomes^54^.

Targets that were identified as consistently regulated by TGF-β at transcript and protein levels in both young and senescent cells included TGFBI (up-regulated) and SEMA3B (down-regulated). TGFBI protein is secreted and deposited in extracellular matrix^55,56^ By interacting with integrins, it mediates cell adhesion and migration^57–59^ and thus may regulate tumour growth and angiogenesis^60^. SEMA3B is also a known regulator of angiogenesis acting to inhibit competitively VEGF signalling pathway^61^. Since the peritoneal mesothelium is a well-established source of angiogenic activity in the peritoneum^62^ and TGF-β is a potent inducer of VEGF in HPMCs^48^, it remains to be determined how the alterations in TGFBI and SEMA3B expression contribute to changes in the angiogenic potential in the aging mesothelium.

An important limitation of this study is that all parameters were assessed at a single time point. Because changes in gene expression, protein abundance, and cell structure most likely follow different time-dependent patterns, future experiments need to assess these parameters at various times after TGF-β stimulation.

**Figure 8:**
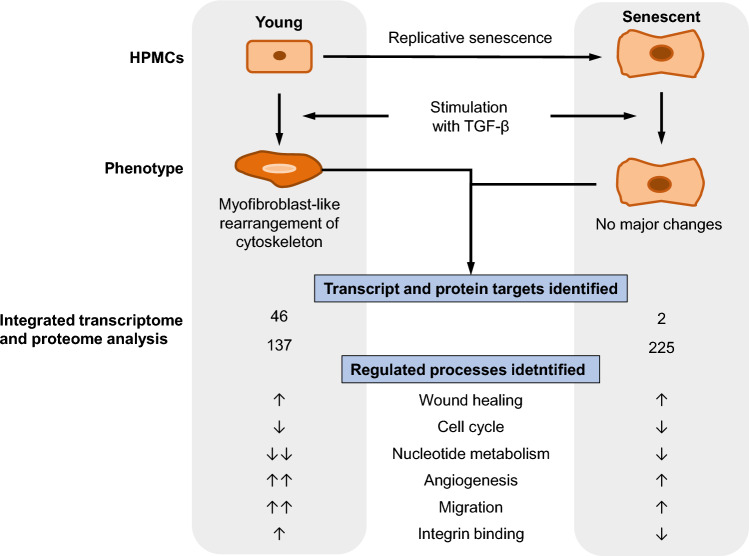
Graphical summary of changes observed in young and senescent HPMCs upon TGF-β stimulation. Omentum-derived HPMCs were grown to senescence. Young and senescent cells from the same donors were then treated in parallel with TGF-β1 (1 ng/ml) for 72 h. After the exposure, cells were analyzed for ultrastructure, and transcriptome and proteome profiles. Responses to TGF- β identified both at the transcript and protein levels are shown. Arrows reflect up- and down-regulated processes, with the number of arrows corresponding to the magnitude of changes observed.

In conclusion, the profound morphological changes seen in senescent HPMCs are not significantly exacerbated by TGF-β. Likewise, changes in global gene and protein profiles in response to TGF-β appear to be less pronounced in senescent cells (Fig. 8). Among the genes and proteins affected by TGF-β, those involved in angiogenesis and fibrosis deserve further attention as they may contribute to adverse peritoneal remodelling in disease.

## Methods

### Ethical approval

The study received ethical approval from the Bioethics Committee of the Poznan University of Medical Sciences (# 2022-569) for the use of discarded omental tissue for research purposes and all patients gave their informed consent. This study was conducted in accordance with relevant guidelines and regulations.

### Cell culture

HPMCs were obtained from pieces of the greater omentum removed during elective abdominal surgery from consenting patients. HPMCs were isolated, identified, and cultured essentially as previously described^48^. HPMCs were cultured for 7–9 passages before entering senescence^63^. Cells were regarded as senescent when proliferation ceased and > 70% cells stained for senescence-associated β-galactosidase (SA-β-Gal), as determined using the SA-β-Gal Staining Kit (Cell Signaling Technology, Danvers, MA, USA). For TGF-β stimulation, paired cultures of young and senescent HPMCs obtained always from the same donor were first deprived of foetal calf serum (FCS) for 24 h and then treated for 72 h with 1 ng/mL of human recombinant TGF-β1 (R&D Systems, Minneapolis, MN, USA) in medium containing 0.1% FCS to maintain basic cell viability. As previously shown, under these conditions, TGF-β1 can effectively induce MMT in HPMCs^10,32^.

### Immunofluorescence

Cells were fixed with 3.7% paraformaldehyde, permeabilized with 0.1% Triton X-100, blocked with 1% bovine serum albumin (BSA), and incubated for 15 min with 330 pM Alexa Fluor®488 Phalloidin (Cell Signaling Technology, Danvers, MA, USA) in PBS containing 0.1% BSA and 0.05% Tween 20. After that cells were counterstained with 4′,6-diamidino-2-phenylindole (DAPI) (Invitrogen/ThermoFisher Scientific, Waltham, USA) and visualized on Cellinsight CX5 platform (ThermoFisher). Images of untreated and TGF-β-treated cells (n = 6 for each condition in young cells and n = 3 for each condition in senescent cells) were captured at 200 × magnification and phalloidin staining intensity was quantified and normalized to nuclear staining with ImageJ software. To assess the effect of TGF-β and senescence, the data were analyzed by two-way ANOVA.

### Electron microscopy

Cells were harvested with a 0.05% trypsin/0.02% EDTA solution, washed with PBS and fixed with 2.5% glutaraldehyde in PBS for 1 h at 4 °C. After that, cells were post-fixed with 1% osmium tetroxide for 30 min at room temperature, washed, dehydrated in a graded series of ethanol (40–100%), and infiltrated and embedded in Epon epoxy resin (Plano, Marburg, Germany). Ultrathin (40–60 nm) sections were mounted on copper grids, contrasted with 5% uranyl acetate in 50% ethanol (30 min) and 1% lead citrate in water (30 min), and analysed with a Jeol transmission electron microscope at 80 kV (Jeol, Tokyo, Japan).

### Microarray expression study

The microarray study was conducted according to previously described procedures^64–66^. Total RNA from HPMCs was extracted with TRI Reagent (SigmaAlrdich, Merck Life Science, Darmstadt, Germany) purified with the Quick-RNA MiniPrep kit (Zymo Research Corp., Irvine, CA, USA) and quantified by spectrophotometry. 100 ng of total RNA was subjected to transcription in vitro, biotin labelling, and cDNA fragmentation using Affymetrix GeneChip® WT Plus Reagent Kit (Affymetrix, Santa Clara, CA, USA). The biotin-labelled cDNA fragments (5.5 μg) were hybridised with the Affymetrix® Human Gene 2.1 ST Array Strip (Affymetrix, Santa Clara, CA, USA) together with control cDNA and oligo B2. The hybridisation process was conducted with the AccuBlockTM Digital Dry Bath hybridisation oven (Labnet International, Inc., Edison, NJ, USA) at 45 °C for 20 h. After hybridisation, the microarrays were washed and stained by the Imaging Station from a Gene Atlas System (Affymetrix, Santa Clara, CA, USA). Preliminary assessment of the scanned strips was conducted using Affymetrix Gene Atlas TM Operating Software (Affymetrix, Santa Clara, CA, USA). The quality of gene expression data was verified according to software-specific quality control criteria.

### Microarray data analysis

The CEL files obtained from microarray scanning were used in further analyses using a BioConductor repository with the relevant Bioconductor libraries of the statistical R programming language (v4.1.2; R Core Team 2021). A robust multiarray average (RMA) algorithm integrated within the “Affy” library was applied to normalise and compute the expression values of examined genes^67^. The gene data table was formed by merging the annotated data table from the BioConductor “oligo” package with the normalised expression dataset^68^. Genes exhibiting low variance were removed using a variance-based filtering function from the “genefilter” library^69^. Expression data of the top 500 genes with the highest variance were utilised for principal component analysis (PCA) using the “factoextra” library^70^. Differential expression analysis and statistical assessment were performed with the “limma” library, employing linear models designed for microarray data^71^. Differentially expressed genes were determined using criteria involving an absolute fold difference exceeding 2 and a p-value of less than 0.05 with a 25% false discovery rate (FDR) correction. The general profile of transcriptome regulation was illustrated as a volcano plot, showing the total number of up-and downregulated genes. Obtained results were visualised using “ggplot2” and “ggprism” libraries^72,73^.

### Assignment of differentially expressed genes to relevant Gene Ontology (GO) Terms

For each analysed comparison, up and down-regulated genes were subjected separately to functional annotation and clusterisation using the DAVID (Database for Annotation, Visualisation, and Integrated Discovery) bioinformatics tool^74^. Entrez IDs of differentially expressed genes were uploaded to DAVID by the “RDAVIDWebService” BioConductor library^75^, where DEGs were assigned to relevant GO terms, with subsequent selection of significantly enriched GO terms from GO biological process (BP), cellular component (CC) and molecular function (MF) databases. Using the same approach, an enrichment analysis was carried out for differentially expressed genes in relation to Kyoto Encyclopedia of Genes and Genomes KEGG signalling pathways^76–78^. The p-values of selected GO terms were corrected using Benjamini–Hochberg correction described as adjusted p-values. Relevant GO ontological groups with adjusted p-values below 0.05 were visualised using a bubble plot. Detailed analysis of genes belonging to selected ontological groups, with their expression fold changes, are presented as heatmap using “ComplexHeatmap” library^79^.

### Gene set enrichment analysis (GSEA)

GSEA was used to determine potential enrichment or depletion in gene expression between two distinct biological cohorts. This approach employed predefined gene sets encompassing Gene Ontology (GO) terms and pathways. The method uses the Kolmogorov–Smirnov (K-S) statistical test to identify significantly enriched or depleted groups of genes. GSEA analysis was performed using the FGSEA library^80^. Normalised fold change values of all genes were log2 transformed and ordered. The enrichment of gene sets was examined in relation to the Reactome database (Molecular Signatures Database)^81^. Genes belonging to the selected set were ranked according to the difference in their expression level using a signal-to-noise ratio with 10,000-time permutation. By walking down, the ranked list of genes, the enrichment score (ES) was calculated for each selected gene set^80^. These scores (ES) were normalised by their gene set size, and FDR corrected false positives. The top twenty significantly enriched and depleted ontological terms (with the highest and lowest normalised enrichment score—NES) were visualised as bar plots.

### Protein extraction

To isolate cell proteins, HPMCs were washed extensively with ice-cold PBS, lysed with RIPA buffer with supplements (50 mM Tris, pH 7.4, 420 mM NaCl, 1% NP-40 (IGEPAL CA-630), 0.25% sodium deoxycholate, protease inhibitors (cOmplete, EDTA-free Protease Inhibitor Cocktail, Roche Diagnostics, Basel, Switzerland), 250 mM sodium fluoride, 50 mM sodium orthovanadate) and cleared by centrifugation. Total protein concentration was determined with the Bradford protein assay (Bio-Rad Protein Assay Dye Reagent, Bio-Rad Laboratories, Hercules, CA, USA) and the samples were stored at -80 °C until assayed.

### Proteomics analysis

The whole proteomics analysis was conducted as previously described^82^. In brief, 70 µg total protein of each sample and an internal pooled standard (IPS), consisting of equal parts of all samples, were used. Digestion was performed using single-pot, solid-phase enhanced sample preparation (SP3). All samples were reduced (10 mM dithiothreitol for 1 h at 56 °C), alkylated (55 mM 2-iodoacetamide, 30 min at RT), and proteins were bound to SP3 beads (10:1 beads:protein ratio, GE Healthcare, Chicago, IL, USA), washed with 80% ethanol and acetonitrile, and subjected to on-bead digestion with trypsin/LysC (1:25 protease:protein ratio, Promega, Madison, WI, USA) overnight at 37 °C in 50 mM ammonium bicarbonate, pH 8.5 (SigmaAlrdich, Merck Life Science, Darmstadt, Germany). After elution peptides were desalted (Pierce Peptide Desalting Columns, ThermoFisher Scientific); dried in a vacuum concentrator, and reconstituted in 100 mM tetraethylammonium bromide (TEAB), pH 8.5 (SigmaAlrdich, Merck Life Science). Peptide concentration was determined according to the manufacturers’ protocol (Colorimetric Peptide Assay, ThermoFisher Scientific).

For multiplexing, peptide labelling with isobaric tandem mass tags (TMTpro, ThermoFisher Scientific) was performed according the manufacturers’ protocol. TMTpro reagents were reconstituted with acetonitrile and 25 µg per sample were labelled with TMTpro. After incubation for 1 h at RT the reaction was quenched by addition of 5% hydroxylamine (SigmaAlrdich, Merck Life Science) in TEAB and incubation for 15 min at RT. Labelling efficiency was determined via LC–MS.

Pooled samples (2 × 12 samples + IPS) were concentrated and desalted (Pierce Peptide Desalting Columns, ThermoFisher). Eluates were dried in a vacuum concentrator and reconstituted in 20 mM ammonia formate buffer, pH 10 before fractionation at basic pH. Two-dimensional liquid chromatography (LC) was performed by reverse-phase chromatography at high and low pH. In the first dimension peptides were separated on a Gemini-NX C18 (150 × 2 mm, 3 µm, 110 A, Phenomenex, Torrance, USA) in 20 mM ammonia formate buffer, pH 10 and eluted over a 48 min gradient from 0 to 60% solvent B followed by 5 min at 100% solvent B at 50 µl/min using an Ultimate 3000 RSLC micro system (ThermoFisher Scientific) equipped with a fraction collector. Thirty-six fractions were collected in a time-based manner (every 30 s from min 11.5 to 57). Organic solvent was removed in a vacuum concentrator and samples were reconstituted in 0.1% trifluoroacetic acid.

Fractions were analysed on an Ultimate 3000 RSLC nano coupled directly to an Exploris 480 with FAIMSpro (all Thermo Fisher Scientific). Samples were injected onto a reversed-phase C18 column (50 cm × 75 µm i.d., packed in-house) and eluted with a gradient of 4% to 38% mobile phase B over 94 min by applying a flow rate of 230 nl/min. MS scans were performed in the range from *m*/*z* 375–1650 at a resolution of 120,000 (at *m*/*z* = 200). MS/MS scans were performed choosing a resolution of 30,000 with the turboTMT mode for TMTpro Reagent; normalized collision energy of 33%; isolation width of 0.7 m*/z* and dynamic exclusion of 90 s. Two different FAIMS voltages were applied (− 40 V and − 60 V) with a cycle time of 1.5 s per voltage. FAIMS was operated in standard resolution mode with a static carrier gas flow of 4.6 L/min.

The acquired raw MS data files were processed and analysed using Proteome Discoverer (v2.4.0.305, Thermo Fisher). SequestHT was used as search engine and following parameters were chosen: database: Homo sapiens (SwissProt, downloaded on 2021-09-24); enzyme: trypsin; max. missed cleavage sites: 2; static modifications: TMTpro (K and peptide N-terminus) and carbamidomethyl (C); dynamic modifications: oxidation (M), deamidation (N, Q), acetyl (protein N-terminus), Met-loss (M) and Met-loss + Acetyl (M); precursor mass tolerance: 10 ppm; fragment mass tolerance: 0.02 Da. For reporter ion quantification the most intense *m/z* in a 20 ppm window around the theoretical *m/z* was used. Correction of isotopic impurities for reporter ion intensities was applied. Only unique peptides were used for quantification, which was based on S/N values with an average S/N threshold of 10. Normalization was based on total peptide amount and scaling mode on controls average (internal standard). Only peptides and proteins with FDR < 0.01 are reported and single peptide identifications were excluded from the dataset. The two multiplex runs were scaled and normalized via the IPS and combined.

### Statistical analysis and graphical representation

Biological pathway enrichment analysis and functional analysis of differentially abundant proteins was performed using the Protein Analysis Through Evolutionary Relationships (PANTHER) database version 17.0. The PANTHER analysis tool was used to performed enrichment analysis for the identification of over-represented biological pathways by a gene list. Annotation databases included in the analysis were Gene Ontology (GO) cellular component and molecular function. Data and statistical analyses and graphical representations of results were performed using R (v4.0.3; http://www.r-project.org/). Pathway identification by Ingenuity Pathway Analysis (IPA 7.0, Qiagen, http://www.ingenuity.com), their respective predicted up/down regulation patterns and their affections by differentially abundant proteins were calculated for each functional pathway by a one-tailed Fisher’s exact test at an alpha level of 0.05. The IPA calculated z-score assessed the match of observed and predicted up/down regulation patterns and served as a predictor for the activation state. Differential protein abundances between conditions were analysed with linear models for microarray data (LIMMA) using the R package “limma”^71^. Limma is a combinatory statistical approach for large-scale expression studies fitting linear models for each gene/protein and utilizing Empirical Bayes and other shrinkage methods to borrow information across genes/proteins to stabilize the analysis and correct variance by shrinking it towards a pooled variance^71^. As mass spectrometry acquired proteomic data can be noisy, large, hierarchical in nature, and imbalanced due to acquisition and pre-processing methods, LIMMA, although being initially developed for microarray data, displayed superiority over conventional statistical modelling approaches (e.g. generalized linear models^83^. Correction of multiple testing was performed by using the Benjamini–Hochberg procedure.

### Supplementary Information

Supplementary Table 1.

Supplementary Table 2.

Supplementary Table 3.

Supplementary Figures.

## Data Availability

Mass spectrometry data have been deposited into the ProteomeXchange Consortium (http://proteomecentral.proteomexchange.org) via the PRIDE partner repository with dataset identifier PXD048090.
