# Supplementary material for: Effect of cellular senescence on the response of human peritoneal mesothelial cells to TGF-β
Source: Sci Rep. 2024 Jun 3;14:12744. doi: 10.1038/s41598-024-63250-1 (PMC11148043; doi:10.1038/s41598-024-63250-1)
Supplement: Supplementary file 4 — Supplementary Figures. [file 41598_2024_63250_MOESM4_ESM.pdf]

Supplementary Figures

Supplementary Fig.S1.

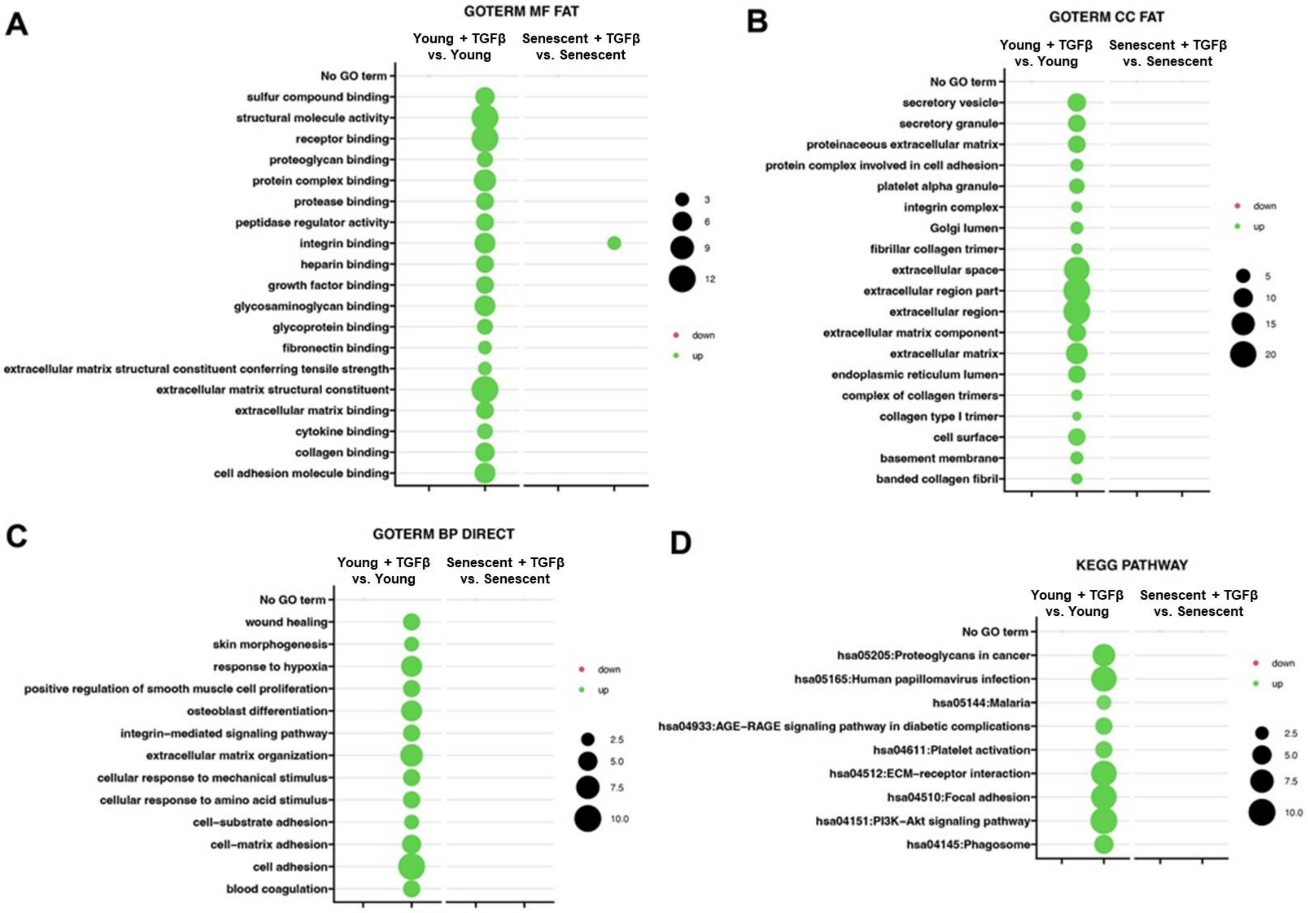

Supplemental. Fig. S1. Bubble plot of enriched gene ontological (GO) terms by significantly up- or down-regulated genes based on: GOTERM MF FAT (**A**), GO CC FAT (**B**), GO BP DIRECT (**C**) and KEGG PATHWAY (**D**) annotations databases. The graph shows only the GO groups above the established cut-off criteria (*p* with multiple testing correction <0.05). The size of each bubble reflects the number of differentially expressed genes assigned to the GO BP terms, while the intensity of the bubble's transparency displays a *p*-value (more transparent indicates closer to the *p*=0.05 cut-off value). The green bubble indicates stimulated GO terms, and the red bubbles - inhibited.

Supplementary Fig.S2.

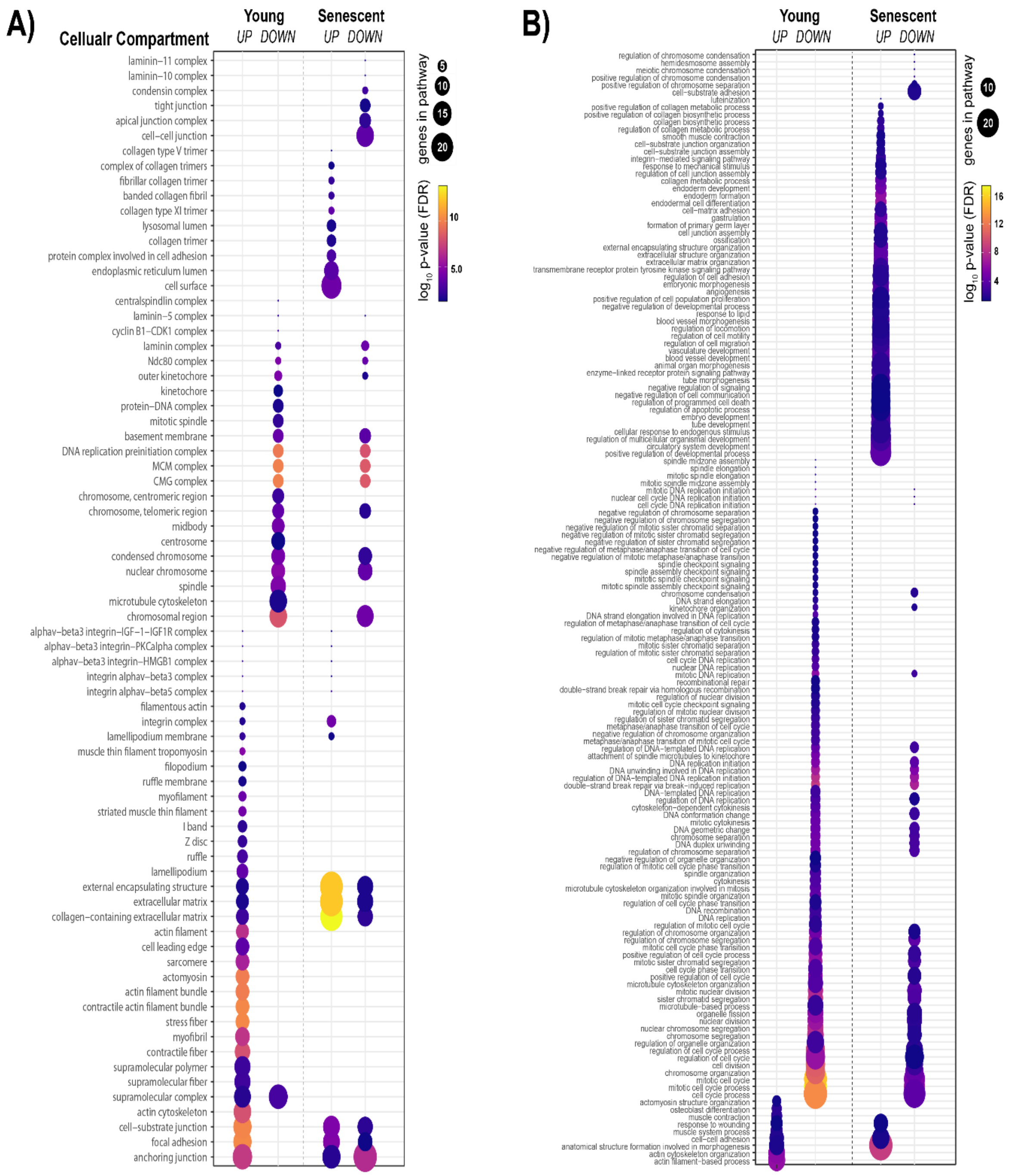

Supplemental. Fig. S2. Bubble plot of pathway enrichment analysis of proteins significantly regulated after TGFβ stimulation. (**A**) Regulation of molecular compartment. (**B**) Regulation of biological processes.
